# The Interdisciplinary Management of Lung Cancer in the European Community

**DOI:** 10.3390/jcm11154326

**Published:** 2022-07-26

**Authors:** Luca Bertolaccini, Shehab Mohamed, Claudia Bardoni, Giorgio Lo Iacono, Antonio Mazzella, Juliana Guarize, Lorenzo Spaggiari

**Affiliations:** 1 Department of Thoracic Surgery, IEO, European Institute of Oncology IRCCS, 20141 Milan, Italy; mohamed.shehab@ieo.it (S.M.); claudia.bardoni@ieo.it (C.B.); giorgio.loiacono@ieo.it (G.L.I.); antonio.mazzella@ieo.it (A.M.); lorenzo.spaggiari@ieo.it (L.S.); 2 Unit of Interventional Pulmonology, IEO, European Institute of Oncology IRCCS, 20141 Milan, Italy; juliana.guarize@ieo.it; 3 Department of Oncology and Hemato-Oncology, University of Milan, 20141 Milan, Italy

**Keywords:** lung cancer, multidisciplinary management, tailored therapy

## Abstract

Lung cancer continues to be the largest cause of cancer-related mortality among men and women globally, accounting for around 27% of all cancer-related deaths. Recent advances in lung cancer medicines, particularly for non-small-cell lung cancer (NSCLC), have increased the need for multidisciplinary disease care, thereby enhancing patient outcomes and quality of life. Different studies in the European community have evaluated the impact of multidisciplinary care on outcomes for lung cancer patients, including its impact on survival, adherence to guideline treatment, utilization of all treatment modalities, timeliness of treatment, patient satisfaction, quality of life, and referral to palliative care. This publication will examine the roles and duties of all multidisciplinary members and the influence of multidisciplinary care on lung cancer outcomes in Europe. Multidisciplinary treatment is the foundation of lung cancer treatment. The optimal setting for interdisciplinary collaboration between specialists with complementary functions is multidisciplinary meetings. Multidisciplinary care in lung cancer facilitates the delivery of a high-quality service, which may improve lung cancer patients’ survival, utilization of all treatment modalities, adherence to guideline management, and quality of life, despite the fact that only limited observational data have demonstrated these results. To confirm the relationship between multidisciplinary treatment and improved lung cancer patient outcomes, however, further research is required.

## 1. Introduction

Lung cancer remains the leading cause of cancer-related mortality among men and women worldwide, accounting for approximately 27% of all cancer-related deaths. The recent advancements in therapies for lung cancer, especially for non-small-cell lung cancer (NSCLC), have created a greater demand for multidisciplinary management of this disease, improving outcomes and quality of life. Different treatment options have been described, including surgery, radiation therapy, systemic therapy (chemotherapy, targeted therapies, or immunotherapy), or a combination of two or three modalities may be used. When needed, a palliative approach to treatment may be appropriate for patients with advanced disease, significant comorbidities, or poor performance status. Due to these multiple treatment options available, different specialists need efficient collaboration to develop a more specific patient-centred management plan. One of the ten goals regarding multidisciplinary cancer management outlined in the Quality Cancer Care Statement by the American Society of Clinical Oncology (ASCO)—the European Society for Medical Oncology (ESMO) states that the “Optimal treatment of cancer should be provided by a team that includes, where appropriate, multidisciplinary medical expertise composed of medical oncologists, surgical oncologists, radiation oncologists, palliative care experts, as well as oncology nurses and social workers. Patients should also have access to counselling for their psychological, nutritional, and other needs.” [1].

In July 2007, at the Lisbon roundtable held during the Europe meeting, under the Portuguese Presidency, one of the conclusions stated was that a multidisciplinary team approach to lung cancer care is required for European lung cancer centres [2].

Another similar statement was recommended by the American Thoracic Society (ATS) and the European Respiratory Society (ERS), which says that all centres offering thoracic oncology services should have multidisciplinary clinics and a thoracic oncology multidisciplinary meeting [3].

The cooperation of all these figures and a multimodality treatment allowed extended care to patients who previously had no treatment options. Moreover, of course, case discussions among providers of varying backgrounds can positively affect treatment plans. Case presentations between specialists can be discussed under several models of multidisciplinary care delivery, such as a regular multidisciplinary meeting, case conference, or tumour board where everyone attends a regular meeting to present, discuss, and make decisions on patient management. Multidisciplinary meetings can take place as face-to-face or virtual meetings, for example, in the case of COVID-19 restrictions. The impact of multidisciplinary care has been already assessed by different studies in the European community in terms of outcomes for lung cancer patients, including its impact on survival; on adherence to guideline treatment; on utilisation of all treatment modalities; on the timeliness of treatment; on patient satisfaction; and on quality of life and referral to palliative care. This manuscript will review the role and responsibilities of all the multidisciplinary members and the impact of multidisciplinary care on lung cancer outcomes in the European community.

## 2. Interdisciplinary Service

An active multidisciplinary lung cancer service is an essential aspect of multidisciplinary lung cancer care, with particular attention to patient-focused care and an attempt to improve the patient journey through collaboration, communication, and streamlining of diagnostics and treatment [4]. In order to have an efficient multidisciplinary lung cancer service, interested physicians should be achieved amongst members of the hospital or community healthcare team with relevant expertise. The interdisciplinary tumour board members must attend meetings frequently to identify lung cancer patients that could benefit from multidisciplinary evaluation and management [5]. Inappropriate work scheduling and time management can compromise physicians’ attendance at multidisciplinary meetings. They can impact the quality of multidisciplinary discussions, and this can happen, for example, through daily clinical ward duties.

For this reason, all members should have dedicated time for multidisciplinary meetings. All other activities should be planned appropriately, including additional time to cover administrative duties that may arise from tumour boards [6,7]. To ensure tumour board efficiency, a competent multidisciplinary meeting lead physician who is an established clinician in the hospital from any speciality among those in the team must be defined. The team leader must obtain opinions from different specialists to assess the meeting’s overall quality and to provide documentation of adequate management plans [8,9,10]. The leader and the members should work closely to optimise patient management and avoid service disruption. Limits to an efficient multidisciplinary meeting can be poor leadership, insufficient teamwork, and time pressure [11,12]. In order to solve this issue, thoracic oncology training in Europe to ensure competent candidates was introduced by the European Respiratory Society as HERMES (Harmonising Education in Respiratory Medicine for European Specialists) European Curriculum recommendations. This program shall train efficient thoracic oncology leads enrolled in any speciality [13,14]. Generally, communication has a crucial role in the smooth running of the entire work, either for the relationship between tumour board members and between clinicians and the patient, the family, and the caregivers, and specific communications skills training is required to explain clearly the different steps of the treatment journey [15]. Consultation and a final agreement with all tumour board members are advised before a treatment plan, considering the complex and increasing rate of multimodality therapy. The quality of the discussion in the multidisciplinary team may be influenced by several factors, such as poor leadership and lack of time and information technology (IT) support, which may be essential, especially in many cases. In the last two years, due to the recent coronavirus disease 2019 (COVID-19) pandemic, multidisciplinary meetings have become gradually virtual to avoid direct contact between multiple clinicians to contain the spread of contagion. For this reason, virtual multidisciplinary team meetings became the standard of care to review patient cases at a safe distance. In this view, a lack of resources about information technology may slow the tumour board meeting due to an incorrect projection of the exams and pathology results on the screen [16].

For this latter, ESMO has published new guidelines and recommendations on managing and treating lung cancer patients in the era of COVID-19 [17].

## 3. Multidisciplinary Meeting Roles and Responsibilities

Multidisciplinary meetings include clinicians and other health professionals. Clinicians involved in lung cancer multidisciplinary meetings should include a thoracic surgeon, radiation oncologist, medical oncologist, respiratory physician, pathologist, nuclear medicine physician, radiologist, and palliative care physician [8,18,19,20]. Other tumour board members could include interventional pulmonologists, clinical nurse specialists, MDM coordinators, psychologists, clinical trials coordinators, nutritionists, physical and occupational therapists, trainees and medical students. Every meeting member should attend on schedule most of the meetings. At least one team member should know the patient case and be present for the discussion and decision-making process.

**Figure 1:**
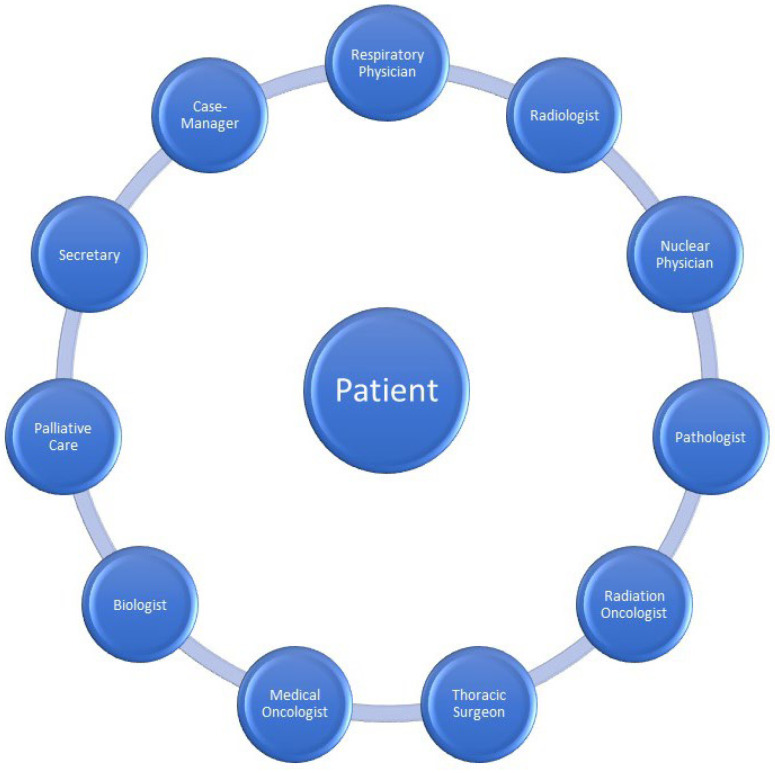
All the specialists contribute to the interdisciplinary management of lung cancer.

Moreover, to ensure diversity of opinions and optimal decisions, at least a minimum of one attending member from each speciality is necessary. In case of attendance is not possible, arrangements should be made to ensure that all decisions are made considering all required specialities. Additionally, late multidisciplinary meeting case additions should be avoided unless clinically urgent. This can provide insufficient time to prepare these cases [19,20]. The frequency of multidisciplinary meetings varies between institutions. At least one multidisciplinary meeting takes place weekly in high-volume centres, unlike small institutions where MDM may not be hosted regularly due to a small number of specialists, especially for rare tumours such as mesothelioma or sarcomas. An irregular frequency of multidisciplinary meetings is associated with more comprehensive time management for definitive treatment. The frequency of tumour boards is essential, especially for rapidly progressive neoplasms such as small-cell lung cancer. There should be a rapid referral to the oncologists for immediate treatment and management without waiting for a multidisciplinary meeting. In this group of patients, the specialists must make a quick decision in the best interest because the risk of missing the right time to be treated is higher than waiting for a potential multidisciplinary meeting [19,20]. Administrative support by the MDM coordinator plays an essential role in a functional and efficient multidisciplinary meeting. In the following sections, we will explain the crucial roles of the different multidisciplinary team members (Figure 1).

### 3.1. Respiratory Physician

Respiratory physicians play a crucial role in multidisciplinary lung cancer discussions. These clinicians are involved in lung cancer patient screening, prevention, risk factor evaluation, diagnosis, management, and follow-up. Patients who would benefit from smoking cessation are identified and referred to the appropriate services for assessment and management, which may occur prior to the multidisciplinary conference [21]. The majority of the time in the European Community, respiratory physicians interested in lung cancer are the first clinicians recommended for patients with a lung cancer suspicion, leading the diagnostic process with medical oncologists [3]. Interventional pulmonologists play a crucial role as members of the MDM. They must be physically present at the multidisciplinary meeting in order to select patients and other professionals, such as thoracic surgeons, who may perform interventional procedures for lung cancer staging, treatment, or palliative care. In certain patients, palliation can be achieved through airway stenting or debulking, offering immediate relief from airway blockage and, in certain situations, enabling future definitive oncological treatments, particularly for patients who have relapsed in later phases of treatment. Pulmonologists should discuss and choose patients during multidisciplinary meetings following a complete airway assessment, consideration of the underlying disease and its natural history, comorbidities, and the patient’s performance status [22].

### 3.2. Medical Oncologists

Medical oncologists are core members of the multidisciplinary meeting. They have special training in diagnosing and treating lung cancer, offering personalised systemic management and using chemotherapy, biological therapy, and targeted therapy to prolong survival and improve quality of life. The predominant role of this specialist is to identify the most appropriate drug regimen, considering the treatment’s toxicity, safety, and cost-effectiveness [23]. Moreover, the specialist can refer the patient to new drugs or clinical trials if available. Medical oncologists can also provide input for supportive care for those with late complications of oncological therapy and advanced stage when palliation may be indicated. To administer the most specific and tailored cancer therapy, medical oncologists contribute through a management plan to achieve cancer diagnosis through detailed cancer characterisation and molecular analysis techniques [24]. They are also involved in the management of patients with poor performance status, the elderly, patients with comorbidities, adolescents, and pregnant women. To identify these particular groups of patients and enrol them in new clinical trials providing access to personalised therapies, radiation oncologists, respiratory clinicians specialised in thoracic oncology, medical oncologists, and pathologists may organise a separate multidisciplinary tumour board [25].

### 3.3. Radiation Oncologist

During the lung cancer multidisciplinary meeting, the radiation oncologist collaborates with the thoracic surgeon, the medical oncologist, and the respiratory physician to provide the most effective treatment. The majority of the time, this professional must treat patients with a poor response to previous treatments or disease progression [26]. The radiation therapy technique, such as volumetric-modulated arc therapy (VMAT), intensity-modulated radiation therapy (IMRT), or stereotactic ablative body radiotherapy (SABR), must be chosen appropriately, taking into account the patient’s efficacy, potential adverse effects, and clinical status. The radiation oncologist must routinely assess the patient’s and tumor’s clinical conditions in order to determine the most effective diagnostic and staging protocols and radiation therapy strategy, radical as a treatment for people with early-stage lung cancer who are unable to undergo surgery. Comorbidities and potential dangers of patients must be taken into account, particularly in the case of combined therapies such as chemotherapy and radiation therapy. Protecting healthy tissues and important organs such as the lungs, heart, and liver from radiation therapy is essential [26].

### 3.4. Thoracic Surgeon

The impact of surgeon volume and specialization in thoracic surgery has been shown to be a beneficial factor in lung cancer surgery 30-day mortality [27]. Thoracic surgeons participating in multidisciplinary lung cancer conferences must be familiar with the case and thoracic disorders in order to identify surgical treatment candidates. During the multidisciplinary conference, this specialist will also be able to identify patients who could benefit from surgical resection, the need for optimisation of their comorbidities ahead to surgery, or the necessity for a specific therapy (neoadjuvant). On the other hand, the thoracic surgeon must be well-versed in nonsurgical treatments, such as chemotherapy and radiation therapy. These various therapeutic techniques are significant for locally advanced lung cancer and can provide essential inputs for the establishment of thoracic oncology clinical trials [28].

### 3.5. Palliative Care

The role of the palliative care specialist in the lung cancer multidisciplinary meeting is pivotal for the early identification of patients that can have any advantages from palliative therapy to relieve their symptoms related to the disease, such as pain, dyspnoea, insomnia, and gastrointestinal disorders and also from involvement in palliative community services or hospices based on patients’ needs. The latter is also essential for their early involvement in the decision-making of their management [29]. Early palliative care has significantly improved patients’ quality of life and mood [30]. Moreover, pain related to bone metastasis can be alleviated by a radiotherapist, who has a complementary role to a palliative care specialist in this case scenario.

### 3.6. Radiologists and Nuclear Medicine Physicians

In multidisciplinary meetings, radiological and nuclear medicine examinations provide an accurate staging and evaluation of lung cancer. Chest radiologists and nuclear medicine specialists play a vital part in the interpretation process. Radiological exams, such as radiography, computed tomography (CT), and magnetic resonance imaging, and nuclear medicine exams, such as positron emission tomography computed tomography (PET-CT), and bone scintigraphy, must be evaluated thoroughly during the multidisciplinary meeting discussion regarding the optimal management plan [31,32].

### 3.7. Pathologist

In interdisciplinary meetings, radiological and nuclear medicine exams accurately determine the stage and severity of lung cancer. A specialized chest radiologist and nuclear medicine experts play a crucial part in the interpretation process. Radiological exams, such as radiography, computed tomography (CT), and magnetic resonance imaging, and nuclear medicine exams, such as positron emission tomography computed tomography (PET-CT), and bone scintigraphy, must be assessed thoroughly during the multidisciplinary meeting discussion regarding the optimal management plan [31,32].

### 3.8. Biologist

Examining features of successful transdisciplinary research, transparent and straightforward communication serves as a unifying theme. All stakeholders involved in cross-disciplinary collaborations must expand their personal, professional, and interpersonal learning beyond their customary comfort zone. Biologist–physician collaborations can expand the boundaries of interdisciplinary meetings. Despite such collaborations’ undeniable promise and value, sustaining them requires a continual communal effort. Bridge scientists can aid in the development of such projects and partnerships, but funding and institutional support for these roles must be increased.

### 3.9. Clinical Nurse Specialist

Clinical nurse specialists serve as the patient’s representation throughout their management, treatment plan, and follow-up [27,28]. They play a crucial role in multidisciplinary meetings. In the multidisciplinary meeting, they assist with patient needs during the decision-making and treatment planning processes. In addition, they deal with patient assessment, health needs assessment, patient and family education, direct and palliative care, treatment planning, and side effect control. They maintain good communication between the physicians and the patient by providing emotional support, for instance. In addition, they detect new patient needs and encourage referrals to other healthcare and social agencies [33]. This specialist has a thorough understanding of patients’ treatment plans and potential side effects, allowing them to provide patients and their families with correct guidance on how to manage them.

### 3.10. Psychologist

The psychologist should also attend a multidisciplinary meeting or at least be available to provide direct access to psychology services when requested [8,18,19,20]. The role of this specialist is significant and very challenging, from the diagnosis of the disease to the end of the patient’s life through specific tools applied in each stage [34]. The psychologist’s assessment can be divided into two phases: the assessment for any pre-existing mental health issues such as personality traits or attachments and the assessment of illness-related psychological discomfort [35]. This assessment should be considered during the multidisciplinary meeting to inform the patient about the management and treatment plan taking into account the psychological background of the patient. There must be trust and meaningful communication between the psychologist and the patient to identify the patient’s needs and organise a plan [36].

Here we review the primary European studies that examine the impact of multidisciplinary meetings on outcomes such as survival, treatment utilisation, surgical utilisation, radiation therapy utilisation, chemotherapy utilisation, adherence to guideline treatment, timeliness of care, palliative care, quality of life, and patient satisfaction.

## 4. Impact on Survival

In the European community, the influence of multidisciplinary care on lung cancer survival has been recorded in very few single-centre retrospective studies. Price et al. (2002) and Forrest et al. (2005) [37,38] were the first groups from Scotland and the United Kingdom to publish studies nearly twenty years ago. Price et al. published a retrospective analysis of 542 NSCLC patients aged 70 and older. A total of 262 were treated without a multidisciplinary meeting, while 280 were addressed prior to a specific treatment at a multidisciplinary meeting (MDM). The MDMs included three specialized respiratory oncologists three times every week. In this study, they demonstrated a statistically significant improvement in 1-year survival from 18.3 percent to 23.5 percent (*p* = 0.049) of elderly NSCLC patients over the age of 70 referred for radiotherapy following the establishment of an MDM, as well as an increase in the rate of curative radiotherapy and a decrease in the rate of palliative thoracic radiotherapy [39].

In contrast, Forrest et al. conducted a retrospective research at a single institution with 243 inoperable NSCLC (stage III/IV) patients, of which 156 were addressed before to a specific treatment in an MDM and 167 were treated without an MDM. At a single tertiary hospital, the MDMs have included two respiratory physicians, two surgeons, a medical oncologist, a clinical oncologist, a palliative care physician, a radiologist, and a lung cancer nurse. In this study, this group found a statistically significant improvement in the median survival of MDM patients who received increasing rates of active treatment and chemotherapy, but not radiotherapy with curative or palliative purpose [37]. In a more recent retrospective cohort analysis from an Italian group, Tamburini et al. reported a comparable outcome based on 477 NSCLC patients treated with surgery between January 2008 and December 2015, of whom 231 were MDM patients and 246 were seen prior to the establishment of the MDM. A surgeon, medical oncologist, radiation oncologist, nuclear medicine physician, pathologist, radiologist, and lung cancer coordinator attended the MDM’s weekly lung cancer meeting [38]. Tamburini et al. observed a statistically significant improvement in survival after one year for patients with early-stage NSCLC who underwent lung resection, 92 percent in the MDM group against 82 percent in the pre-MDM group [38]. The reported improvement may be attributable to an independent prognostic factor in our study, namely the patients’ comprehensive pre-operative staging. They also found no difference in resection margins, mortality, or postoperative complications.

In contrast, Murray et al. (United Kingdom) published in 2003 a randomized controlled trial in which there was no difference in survival between patients assigned to the two arms, either overall or among those getting curative treatment [40]. From October 1998 to January 2001, 88 patients with probable lung cancer were evaluated in this study, 45 in the central MDM arm and 43 in the traditional arm. A thoracic surgeon, respiratory physicians, medical oncologists, clinical oncologists, palliative care physicians, and a study coordinator were present at MDMs. In addition, there was a trend toward more curative treatment in the MDM arm. The non-cancer diagnosis of about 30% of patients in this trial and the small sample size are crucial limitations that must be acknowledged [40]. According to the stage of cancer, Forrest et al. and Tamburini et al. have studied the impact of multidisciplinary care.

In addition, the same studies have assessed the impact of a given stage on a cohort of patients at all phases. The majority of MDM patients arrived with stage III disease, whereas the majority of non-MDM patients presented with stage IV disease. Forrest et al. showed a statistically significant improvement in the median survival of patients with inoperable stage III/IV NSCLC following the formation of an MDM at a single centre, 6.6 months versus 3.2 months [37,38]. The distribution of these two groups of patients with inoperable stage III and IV disease was comparable, but again, the relatively small number of patients represents a limitation of this study. Neither patient performance status nor co-morbidities were accounted for in this investigation. Considering these few studies, there is little evidence that multidisciplinary meetings have a major impact on lung cancer survival. According to numerous studies, interdisciplinary care significantly improves patients’ survival. However, these investigations are restricted by their retrospective methodology, small sample size, and inability to account for confounding variables that influence the survival of this patient population. Statistical analysis of potential imbalances in prognostic factors between the main two-arm groups has revealed that the group receiving multidisciplinary care has a greater chance of survival.

## 5. Treatment Utilisation

The most crucial purpose of multidisciplinary meetings, the cooperation between different specialised professionals, is to develop an appropriate management plan and determine the most specific treatment for every patient. Murray et al., in their randomised trial, have reported that patients discussed in a multidisciplinary setting are more likely to receive curative treatment, including all treatment modalities. Patients discussed in a multidisciplinary setting recorded twice as likely to have chemotherapy, especially with palliative intent. In this small-size study, there was no significant difference in the use of curative treatment [40]. On the other hand, Forrest et al. stated that a statistically significant number of patients not managed in a multidisciplinary setting were more likely to receive the best supportive care only [37]. This difference may reflect that the non-discussed patients generally have poor performance status with multiple comorbidities, are older, and have more advanced diseases.

### 5.1. Surgery

Study to study, the effect of multidisciplinary meetings on surgical utilization differs. Davison et al. performed a modest retrospective single-institution analysis on 62 patients selected between November 2000 and November 2001, 50 of whom had undergone thoracotomy three years prior to the MDM. In this instance, biweekly interdisciplinary discussions were held by teleconference between a regional centre and a metropolitan tertiary hospital. A thoracic surgeon, respiratory physicians, medical oncologists, clinical oncologists, radiologists, and a lung cancer nurse coordinator were present at MDMs [41]. Patients initially seen in a centre with a department of thoracic surgery experienced a 30% increase in surgery rates after the establishment of a teleconferencing multidisciplinary meeting (MDM) between a regional and tertiary referral centre, presumably because this provided a more direct referral pathway for patients and physicians [42]. In their retrospective observational analysis, Tamburini et al. observed an increase in the frequency of MDM patients receiving mediastinal staging prior to surgery [38]. Overall, individuals having access to thoracic surgery or an established referral channel for thoracic surgeon evaluation who are discussed in a multidisciplinary context have a higher surgical use rate, particularly for stage I and II NSCLC.

### 5.2. Radiation Therapy

In their two retrospective analyses [37,39], Price et al. and Forrest evaluated two categories of radiation-treated patients: curative and palliative. The findings of these trials following multidisciplinary care varied. Forrest et al. [37] examined inoperable NSCLC patients and found comparable rates of curative or palliative radiation therapy before and after establishing an MDM. The sample size of patients, particularly those receiving curative radiation therapy, was however modest. Price et al. discovered a significant increase in curative thoracic radiation therapy and a decrease in palliative thoracic radiation therapy in older patients in a multidisciplinary environment [39] three years earlier. Recent advances in radiation therapy and improved accuracy of radiation delivery have contributed to observed improvements in patient survival over the past decade, making radiation therapy a cornerstone of contemporary lung cancer treatment alongside surgery, chemotherapy, immunotherapy, and targeted therapies. These developments have led to the evolution of radiation therapy into a treatment recommended by guidelines for early-stage and locally progressed lung cancer, but not for small-cell lung cancer. Examples of this enhanced survival include the lowering rates of non-treatment in early-stage lung cancer in population studies and the survival benefits observed in trials including immunotherapy after chemotherapy and radiation therapy in locally advanced NSCLC.

In addition, although radiation therapy has traditionally been utilized for the palliation of advanced lung cancer, it is increasingly being used as a locally ablative treatment for patients with oligometastatic illness. As a result, therapeutic options are getting increasingly complex, and multidisciplinary tumor meetings are assuming an increasingly vital role in the selection of effective methods. With the development of new treatment options, multidisciplinary tumour boards have become essential for selecting and customizing treatment methods, as well as addressing toxicity and survivability concerns.

### 5.3. Targeted Therapies or Immunotherapy

All the reported studies were published before targeted therapies or immunotherapy, so the role of these therapies inside a multidisciplinary meeting was not assessed.

### 5.4. Guideline Treatment

Decisions regarding treating and managing lung cancer patients can be made with the help of different evidence-based clinical practice guidelines, including ESMO and AIOM [17,41]. The few European studies reported in this chapter showed that despite their limitations, such as single institution, retrospective study design, small sample size, and potential referral bias, involving patient case discussion in a multidisciplinary setting is associated with increased utilisation of evidence-based treatment guidelines and greater adherence to guideline-based treatment.

### 5.5. Timeliness of Care

In their two retrospective studies [37,39], Price et al. and Forrest evaluated two categories of radiation therapy patients: curative and palliative. Different outcomes were observed in these trials following multidisciplinary care. Forrest et al. [37] examined inoperable NSCLC patients and found comparable rates of curative or palliative radiation therapy prior to and after the establishment of an MDM. The sample size of patients, particularly those receiving curative radiation therapy, was limited. Three years earlier, Price et al. [39] found a significant increase in the incidence of curative thoracic radiation therapy and a decrease in the rate of palliative thoracic radiation therapy among older patients. In the past decade, the recent advances in radiation therapy and the improved accuracy of radiation delivery have contributed to the observed improvements in patient survival, making radiation therapy, along with surgery, chemotherapy, immunotherapy, and targeted therapies, a cornerstone of modern lung cancer treatment. These advancements have contributed to the evolution of radiation therapy into a treatment recommended by guidelines for early-stage and locally progressed lung cancer, but not for small-cell lung cancer. Examples of this enhanced survival are the declining rates of non-treatment in early-stage lung cancer in population studies and the survival benefits observed in trials including immunotherapy after chemotherapy and radiation therapy in locally advanced NSCLC [43].

In addition, although radiation therapy has long been utilized for the palliation of advanced lung cancer, it increasingly has a function as a locally ablative therapy for patients with oligometastatic illness. As a result, treatment options are becoming increasingly complex, and multidisciplinary tumor conferences are playing an increasingly crucial role in determining the most effective techniques. With the availability of novel treatment options, multidisciplinary tumor boards have become essential for selecting and customizing treatment strategies, as well as controlling toxicity and survivability difficulties [44,45].

### 5.6. Palliative Care, Quality of Life, Patient Satisfaction

Smith et al. reported a qualitative assessment of 497 NSCLC patients visited in the palliative care clinic between January 2009 and January 2011. In a weekly MDM, respiratory physicians, thoracic oncologists, palliative care physicians, lung cancer nurses, and clinical trials discussed their patients’ cases. Patients in this study recognized various benefits of having palliative care services as part of multidisciplinary care, including increased service provision, quicker time for referrals and access to cancer trials, lower transport expenses, and a seamless transition between services [46]. They also observed that collaborative teamwork improves the palliative patient’s experience and is connected with possible cost savings for the organization.

## 6. Discussion

The diagnosis and management of lung cancer, both NSCLC and SCLC, can be very complicated and challenging, and different specialists may be involved; all of this can be time-consuming, especially with new and more specific therapies. Multidisciplinary tumour board meetings involving thoracic surgeons, pulmonologists, oncologists, radiation oncologists, radiologists, dieticians, physiotherapists and palliative care specialists are recommended by ESMO and AIOM and other international organisations for appropriate treatment strategies to simplify this process considerably and to ensure interdisciplinary collaboration to meet patients’ needs. Every specialist described in this chapter has a specific role and responsibilities and can give a complementary contribution to the other specialists in an interdisciplinary setting. Multimodality treatments based on the best available evidence have improved survival and quality of life for patients with lung cancer. Multidisciplinary meetings allow for discussing and reviewing patient cases to determine the most appropriate management and treatment plan in a patient-centred approach. Many studies on the impact of multidisciplinary tumour boards on lung cancer patients have been reported, with different outcomes described. Most of the outcomes are from retrospective studies comparing results before and after multidisciplinary meetings establishment and patients discussed in the multidisciplinary environment compared to patients managed outside this setting. It is difficult to compare these published studies due to different definitions, methods, and outcome measures. Multidisciplinary care appears to have a positive effect on several outcomes in lung cancer despite many limitations, such as many confounders present as heterogeneity of tumour stage, performance status, comorbidities, social and economic status, and access to services, which impact the significance of the findings in the studies. As described above, in the larger cohort trials, there was a significant improvement in survival for patients managed through a multidisciplinary meeting. This setting was also associated with higher rates of utilisation of surgery, chemotherapy, and radiation therapy as curative and palliative treatment. It should also be considered that patients discussed in multidisciplinary meetings are more likely to be younger, with fewer comorbidities, better performance status, and earlier stage disease. For this reason, it is relevant to take into account also the cohort of older patients with comorbidities and lower performance status as the multidisciplinary discussion is necessary to determine the adequate curative treatment, especially for multimodality treatment. Based on the studies considered in this chapter, multidisciplinary care was associated with improved adherence to guidelines and treatments recommendation. The timeliness of treatment for lung cancer has been reported in many studies. However, its relation with multidisciplinary care is complex to assess due to variations in the commencement of the therapy after a potential discussion. A multidisciplinary care setting will likely have a shorter time between meetings and starting treatment. Another important topic is the access to palliative care services that are improved thanks to the presence of a palliative care specialist during the multidisciplinary meeting. This allows all patients needing a palliative care service contribution to be seen promptly [46]. On the other hand, the multidisciplinary discussion may not impact the rate of palliative therapy due to an increased rate of other treatments such as surgery, oncological therapy, or radiation therapy, making the time for palliative care referral longer for this group of patients. Moreover, many limitations have assessed the patient quality of life and satisfaction in a few studies. For this reason, no significant evidence suggests that multidisciplinary care improves patient quality of life. Murray et al. also reported that patients assessed in a multidisciplinary setting are associated with fewer visits to their general physician during their workup and management [40]. For the many limitations described above, ideally, further studies should be done between multiple institutions and with a large prospective design to demonstrate the benefits of multidisciplinary meetings. With all prognostic factors documented, a large cohort study could further examine the impact of survival benefits and treatment use of multidisciplinary meetings. In this setting, the benefits of screening in high-risk populations and the utilisation of newer systemic therapies such as targeted therapies, immunotherapies, and the impact on quality of life and patient-reported outcomes are other outcomes that can be assessed in lung cancer patients. Unfortunately, most institutions already have well-established multidisciplinary processes, and it would be difficult to randomise patients into two main groups, discussed and not discussed at multidisciplinary meetings.

The multidisciplinary discussion has numerous advantages, such as the guarantee of a correct therapeutic decision for the given neoplasm of that patient. Moreover, every doctor is more protected from the error caused by the individual’s opinion (Eminence-Based Medicine). Additionally, the multidisciplinary discussion is a moment of growth for the joining specialists. After more and more discussions, the oncologist learns the surgical indications and the surgeon learns the radiotherapy approach; while discussing with the radiologist, the surgeon could learn to understand a radiological exam, and the radiologist learns the particularities of the image for the surgeon. On these occasions, each specialist brings the updates from his discipline acquired during conventions and conferences and contributes to the multidisciplinary team’s collective growth. The fast and continuous development of medicine and the continuous development of technological innovations increase the difficulties for the individual physician to keep up-to-date and competent. For this purpose, the programs of Continuing Medical Education (CME) were born in all countries of the world. Participating in the CME programs is not a duty for Doctors, as referred to in the national Deontological Codes. However, it is a right for the patients who correctly require careful, up-to-date, and empathic operators. Moreover, this is mainly significant today since patients are increasingly informed about the possible answers of medicine to the different.

## 7. Conclusions

Multidisciplinary care is the cornerstone of lung cancer care. Multidisciplinary meetings are the best setting that ensures close interdisciplinary collaborations between specialists with complementary roles. Although limited observational data have demonstrated these results, multidisciplinary care in lung cancer facilitates the delivery of a high-quality service, which may result in improved survival, utilisation of all treatment modalities, adherence to guideline management, and quality of life for lung cancer patients. However, more evidence is needed to confirm the association between multidisciplinary care and improved lung cancer patient outcomes.
